# Biometric and Emotion Identification: An ECG Compression Based Method

**DOI:** 10.3389/fpsyg.2018.00467

**Published:** 2018-04-04

**Authors:** Susana Brás, Jacqueline H. T. Ferreira, Sandra C. Soares, Armando J. Pinho

**Affiliations:** ^1^IEETA - Institute of Electronics and Informatics Engineering of Aveiro, University of Aveiro, Aveiro, Portugal; ^2^DETI - Department of Electronics, Telecommunications and Informatics, University of Aveiro, Aveiro, Portugal; ^3^Department of Education and Psychology, University of Aveiro, Aveiro, Portugal; ^4^Faculty of Medicine, Institute for Biomedical Imaging and Life Sciences, University of Coimbra, Coimbra, Portugal; ^5^Department of Education and Psychology, CINTESIS-UA, University of Aveiro, Aveiro, Portugal; ^6^Division of Psychology, Department of Clinical Neurosciences, Karolinska Institute, Stockholm, Sweden

**Keywords:** biometrics, emotion, quantization, data compression, Kolmogorov complexity

## Abstract

We present an innovative and robust solution to both biometric and emotion identification using the electrocardiogram (ECG). The ECG represents the electrical signal that comes from the contraction of the heart muscles, indirectly representing the flow of blood inside the heart, it is known to convey a key that allows biometric identification. Moreover, due to its relationship with the nervous system, it also varies as a function of the emotional state. The use of information-theoretic data models, associated with data compression algorithms, allowed to effectively compare ECG records and infer the person identity, as well as emotional state at the time of data collection. The proposed method does not require ECG wave delineation or alignment, which reduces preprocessing error. The method is divided into three steps: (1) conversion of the real-valued ECG record into a symbolic time-series, using a quantization process; (2) conditional compression of the symbolic representation of the ECG, using the symbolic ECG records stored in the database as reference; (3) identification of the ECG record class, using a 1-NN (nearest neighbor) classifier. We obtained over 98% of accuracy in biometric identification, whereas in emotion recognition we attained over 90%. Therefore, the method adequately identify the person, and his/her emotion. Also, the proposed method is flexible and may be adapted to different problems, by the alteration of the templates for training the model.

## 1. Introduction

The recognition of persons and the identification of the corresponding emotions allow a better interaction with computers, systems and environments, enabling an improved user experience (Calvo and D'Mello, 2010). Ludic activities and health care are two obvious application fields, although certainly not the only ones. As an example, we may adapt a game in order to enhance the experience, according to the player state. At home, using the advent of the internet of things, we may think of a house that recognizes the person and understands which radio station he or she prefers, according to the respective emotional state. In therapy, the therapist could rely on the measured emotional state of the patient and adapt the therapy according to the patient needs. In fact, there is a plethora of opportunities where emotion recognition would be beneficial (Calvo and D'Mello, 2010).

The electrocardiogram (ECG) is a well known signal in biomedical applications. Usually, researchers want to reduce the inter-variability that characterizes this kind of signal. However, it is precisely this inter-variability that renders the ECG an interesting signal for biometric systems (Agrafioti and Hatzinakos, 2009). The ECG is not only characterized by inter-variability, but also by intra-variability. Therefore, the biometric models should correctly deal with such variability and find the key that each person carries on the ECG. Notwithstanding this, the intra-variability of the ECG is a rich source of information about the person's state, e.g., allowing emotion identification (Janssen et al., 2013).

### 1.1. ECG biometric identification

Biometric identification refers to methods that use biological data in order to identify someone as being him/herself. There are biometric identification methods using iris, face, fingertip, ECG or electroencephalogram (EEG) (Agrafioti and Hatzinakos, 2009; Odinaka et al., 2012). Some of them are susceptible to falsification, since there is no need of contact or liveliness for acquisition of the signals (Agrafioti and Hatzinakos, 2009). However, our heart is continuously beating and possesses an inter-subject variability that may be used to obtain discriminant information based on the electrical heart characteristics.

There is an increasing interest in using the ECG in biometric identification (Agrafioti and Hatzinakos, 2009; Odinaka et al., 2012; Coutinho et al., 2013). The ECG is subject to changes due to the circadian cycle, or some particular circumstances (e.g., stress, fatigue), conducing to alterations in rhythm and/or amplitude (Odinaka et al., 2012). To overcome this shortcoming, the ECG biometric applications should correctly deal with fluctuations and noise (Agrafioti and Hatzinakos, 2009; Odinaka et al., 2012).

The ECG biometric methods are usually divided in two groups: fiducial and non-fiducial (Odinaka et al., 2012). The fiducial methods use as preprocessing step for heartbeat delineation, the identification of specific points that constitute the ECG (Israel et al., 2005), which is susceptible to processing errors, due to, e.g., noise (Agrafioti and Hatzinakos, 2009; Odinaka et al., 2012). The non-fiducial methods do not need the ECG wave delineation or alignment and, therefore, it is expected that these methods reduce the preprocessing error (Agrafioti and Hatzinakos, 2009; Odinaka et al., 2012). Some methods are only semi-non-fiducial, needing at least the R-peak delineation to align windows (Agrafioti and Hatzinakos, 2009; Odinaka et al., 2012; Carvalho et al., 2017; Pinto et al., 2017). Others are completely non-fiducial (Agrafioti and Hatzinakos, 2009; Fang and Chan, 2009; Sufi and Khalil, 2011; Fang and Chan, 2013) (e.g., methods based on autocorrelation function analysis, which do not require window alignment Agrafioti and Hatzinakos, 2009).

ECG biometric identification implies the collection of ECG data every time the person arrives at the access point. Therefore, and due to the ECG characteristics, there are external variability conditions that affect the signal and its quality, like circadian cycle, or some particular circumstances, that affect the ECG rhythm and/or amplitude (Odinaka et al., 2012). Hence, to grasp the ECG key, the methods should be robust and immune to fluctuations. Also, the system should correctly deal with variability and different sources of noise: muscle, movement, electrodes placement or pathologies (Agrafioti and Hatzinakos, 2009; Odinaka et al., 2012).

In order to obtain a feasible and accurate biometric system, there is a need to implement methods that may correctly deal with traditional problems in biometric identification methods. The application of parameter free methods, such as compression algorithms, have been proved efficient in classification, since there is no pre-assumption about the premises, thus allowing true exploratory data mining (Keogh et al., 2004).

### 1.2. Study purpose

Emotions induce a set of alterations in the cardiovascular response (Agrafioti et al., 2012). However, it is not guaranteed that the response is similar or instantaneous over different physiological systems. In this work, we focus on the cardiovascular response given by the ECG. In the context of biometric identification, the emotional state of a person may be viewed as noise that needs to be removed. However, this is also valuable information about the person individual characteristics, i.e., the ECG is also a source of emotional information, since emotions are accompanied by physiological alterations, namely in the ECG signal.

Assuming the hypothesis that the experience of emotions is idiosyncratic, i.e., that people experience emotions in a unique way (Barrett et al., 2007; Kim and André, 2008; Agrafioti et al., 2012), we propose in this paper a biomarker that is able to both identify the person and his/ her state of emotion. To accomplish this goal, we designed a procedure that works in two sequential steps:

1. First, using the acquired ECG segment, the person is identified (biometric identification).
2. Then, knowing the identity of the person, her/his state of emotion is identified (emotion identification).

In the first step of the procedure (biometric identification), the person is represented by a set of ECG segments belonging to her/him, as varied as possible, i.e., it contains segments of ECG acquired under all available conditions. This variety provides robustness to the biometric identification step. The second step relies on a separation of those ECG segments into classes of interest, in order to create sets (per person) of ECG segments representing the emotion experienced by the person.

In the section 2, we described the methodological part of the paper, including the data description, data collection protocol, and methodological technical details. In section 3, the main results of the work are described and discussed. The conclusion of the work and future directions are presented in section 4.

## 2. Materials and methods

### 2.1. Our approach

In this work, we propose the use of compression methods, which are related to the notion of Kolmogorov complexity, for ECG-based biometric and emotion identification. Therefore, we use parameter free data mining techniques, to overcome most of the problems that affect the traditional ECG-based biometric methods (preprocessing error, biases on ECG segments identification, etc.). These methods allow a true exploratory data mining, since there is no assumption about the premises. They do not need to extract features from the data in order to describe the data. Instead, they evaluate the global data sequence and compare them, in order to find similarities and dependencies. The Kolmogorov complexity of *x*, *K*(*x*), is defined as the minimum size of a program that produces *x* and stops (Li and Vitányi, 2009), where *x* is a string (e.g., of binary digits). A major drawback of the Kolmogorov complexity (or algorithmic entropy) is that it is not computable. To overcome this limitation, it is usually approximated by some computable measure, such as Lempel-Ziv, linguistic or compression-based complexity measures. These approximations provide upper bounds on the true complexity, allowing, e.g., developing similarity/dissimilarity measures (see Pinho et al., 2011; Pratas et al., 2012; Garcia et al., 2013 for recent applications of this concept). This similarity measure is used as a metric to quantify if a new ECG record belongs to a previous known ID group. It does not require the usual preprocessing step that is performed on the ECG, which is wave delineation (Agrafioti and Hatzinakos, 2009; Odinaka et al., 2012). Since the method is based on similarities, computed by data compression methods, the conversion of the real-valued signal to a symbolic time-series is necessary.

The method proposed for emotion identification is based on the idea that, given a database that contains information about participants and their biometric data, we are able to identify the participant. Moreover, we also have information about the different emotional conditions reflected on the ECG response. We use the data to answer the following problems: (1) If we want to identify the participant, we compare the acquired segment with the ECG data that characterizes each participant; (2) If we want to identify the emotion, we look at the data that characterize the emotions of that person. Hence, the ECG data used in both tasks are different. For biometric identification, every instance corresponds to one person, independently of the condition (e.g., emotion). For emotion identification, every instance corresponds to an emotion, associated with the participant.

### 2.2. The ECG

**Figure 1:**
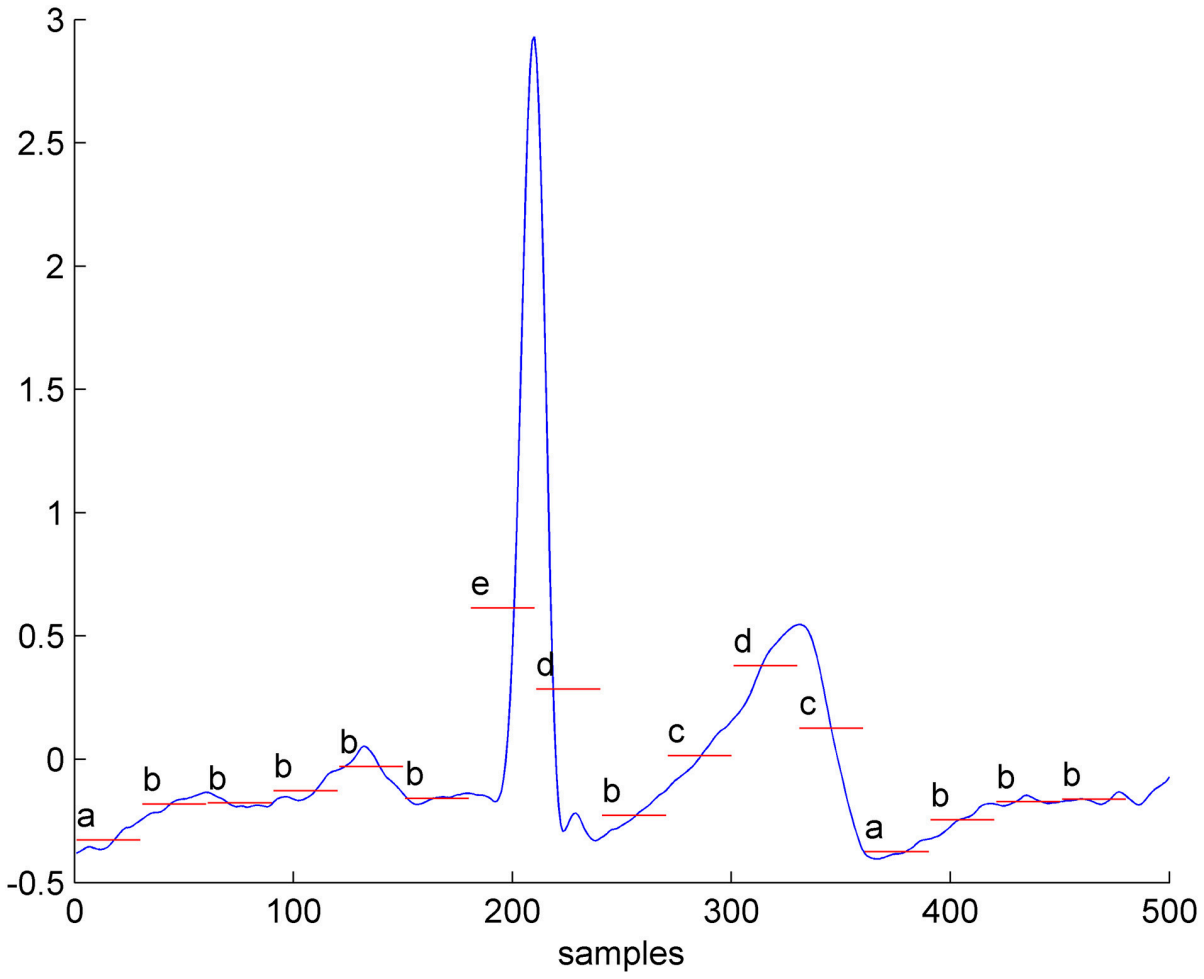
One second of a real-valued ECG converted into the symbolic time series, considering an alphabet size of 6 letters and a window size *w* = 30. The selected parameters in this example are not the optimal for the presented problem, but only selected for method illustration purpose.

The ECG represents the electrical signal that comes from the contraction of the heart muscles, indirectly representing the flow of blood inside the heart, and it is almost periodic. Figure 1 represents a typical ECG wave.

In this work, two ECG databases were used. For the method development and validation, we used a database available online, the PTB database, accessible at the Physionet website (Goldberger et al., 2000). The PTB database was used to evaluate the performance of the proposed method, in comparison with other published works on biometric identification (e.g., Coutinho et al., 2013). Therefore, this validation step allows inferring the performance of the proposed method over other biometric identification methods. This allowed also to understand the feasibility of the method. However, this database does not have data collected in different days, or even subjected to different emotional conditions. Therefore, for a more correct validation, we also tested the method on an ECG database collected at our University, henceforth called emotional database. This database has important different characteristics: longer ECG records; three records per person; each record was influenced by different emotional conditions, enriching the data and hence approximating the used databases to real conditions. Therefore, this database allowed to validate the method:

- In a different database from the one the method was tuned;
- In data collected in different days;
- In data exposed to different external conditions.

The ECG of both databases was collected using pre-gelled electrodes. Although this configuration setup is intrusive (Da Silva et al., 2013), it presents lower noise level. Since the main goal of this paper is to give a proof of concept, i.e., to show that an ECG biometric and emotion identification method based on compression models can correctly identify the person and his emotion, we decided to validate the method in a database with as less as possible external noise. The idea for a future implementation is to collect data with a non-intrusive setup.

#### 2.2.1. The PTB database

The PTB database (Goldberger et al., 2000) is composed by control and unhealthy subjects. However, due to this work goals, we only used the control subjects. The ECG signal was decimated to 500 Hz, using an eighth-order low pass Chebyshev Type I filter with a 200 Hz cutoff frequency. The baseline wander was filtered using a moving average filter with window width of 100 samples. A Butterworth low pass filter of order 10 with a cutoff frequency of 40 Hz was also applied.

In order to select a homogeneous database, we decided to split each participant record in five segments of 20 s, constituting a database of 52 participants and 260 records. Only one record from each participant was considered in the analysis, because not all the participants had more than one record. This procedure was also used in other works (e.g., Coutinho et al., 2013).

#### 2.2.2. The emotional database

For this database, data were collected from 25 healthy participants (10 male), mean age of 22.25 ± 4.21 years, who were not taking medication and had no previous history of psychiatric or psychological disorders. The participants, recruited at the University, gave their written consent and were informed about the possibility of withdrawing from the experiment at any time. The study followed the guidelines of the Declaration of Helsinki and standards of the American Psychological Association.

Each participant watched three movies that induced disgust, fear and neutral emotions, one in each week. From the six basic emotions, which are the most commonly studied (Ekman, 1992), we selected two aversive emotions, fear and disgust (and neutral, as a control emotion). We started by using these two negative emotions, because although they share a similar valence and arousal (e.g., Lang et al., 1999), several studies to date have claimed that they show differences not only in cognitive and behavioral, but also in physiological dimensions (e.g., Cisler et al., 2009). Hence, our goal in this study was to apply the proposed method to investigate whether it allows a successful identification of these negative basic emotions.

The disgust film contained disgusting scenes (Pink Flamingos), the fear film contained horror scenes (The Shining), and the neutral film showed a documentary about the Solar eclipse (Easter Island—Solar Eclipse). Both emotionally active stimulus (disgust and fear) were successfully used to induce disgust and fear in previous studies (e.g., Vianna and Tranel, 2006; de Groot et al., 2012). The duration of each film was 25 min and their presentation order was counterbalanced. The dataset for emotion was collected using the adhesive disposable Ag/AgCl-electrodes Biopac EL503 Ag-AgCl snap electrodes, that were placed following a standard lead II configuration (right arm, left leg and right leg ground; Berntson and Lozano, 2007) and connected to the Biopac MP100, ECG Module (Biopac Systems, Inc.).

Prior to the presentation of each video (disgust, fear, and neutral), the participants were asked to rate their subjective emotions and to watch a 4 min video, displaying a beach sunset with acoustic guitar soundtrack, which served as the resting baseline period. During this time, the participants were instructed to sit quiet and to relax as much as possible. They were also instructed to avoid looking away from the monitor or shut their eyes whenever they found the scenes too distressing. The physiological channels were constantly sampled during the presentation of the videos. Finally, the participants rated the subjective emotions experienced during the presentation of the videos by rating, on 7-point Likert scales (1 = not at all; 7 = very), their self-reported level of disgust, fear, happiness, anger, sadness, surprise, and pleasantness. These results on such measures by these participants showed that the disgust and fear movies, compared to the neutral videos, were successful in inducing the corresponding emotions. Moreover, participants filled in the Portuguese version (Silva and Spielberger, 2007) of the State-Trait Anxiety Inventory (STAI Form Y-1, Spielberger, 1983), as well as the Portuguese version (Galinha and Pais-Ribeiro, 2005) of the Positive and Negative Affect Scale (PANAS; Watson et al., 1988). (A full description of the scales and corresponding results can be consulted on Ferreira et al., 2017.)

In order to assure that the videos were indeed inducing each of the specific emotions, we analyzed subjective ratings from each video presentation. Considering that we are evaluating an interval scale, we used Wilcoxon signed-rank, a non-parametric test, since the data violate an assumption of the normality. The results confirmed our manipulation while showing that in the disgust condition participants reported significantly more disgust than in the fear (*z* = 4.33, *p* < 0.001, *r* = 0.88, where *z* is the statistical value, *p* the significance value, and *r* the effect size of the test), and neutral condition (*z* = 4.38, *p* < 0.001, *r* = 0.89). Moreover, in the fear condition, participants also reported significantly more fear than in the disgust (*z* = 3.69, *p* < 0.001, *r* = 0.74), and neutral conditions (*z* = 4.32, *p* < 0.001, *r* = 0.88). Finally, in the neutral condition, participants reported significantly more happiness than in the disgust (*z* = 3.52, *p* < 0.001, *r* = 0.72), and fear conditions (*z* = 4.11, *p* < 0.001, *r* = 0.82). The acquired ECG was sampled at 1,000 Hz and, for the method application, the ECG was (as in PTB database) decimated to 500 Hz, using the previously described method.

### 2.3. ECG processing

When compression algorithms are used, it is generally preferable the use of symbolic records. The ECG is a real-valued numerical signal, and, in the context of this work, it had to be converted into a symbolic time series. To accomplish this goal, a quantization method was used.

#### 2.3.1. Quantization

The SAX representation (Lin et al., 2002, 2003) was selected as the quantization technique. The performance of the method is dependent on two optimization parameters: (1) The alphabet size; (2) The dimension w of the series segments. It is reported in the literature (Lin et al., 2002, 2003) that there is not a general solution, i.e., it will depend on each particular problem. The method may be summarized as follows:

- The real-valued time series is normalized (by subtraction of the mean value and division by the standard deviation);
- The normalized time series is divided into *N* segments of *w* data points;
- The mean value of each *N*_*i*_ segment matches a symbol in the new data representation.

Figure 1 exemplifies the conversion into symbols of 1 s of ECG data, considering an alphabet size of 6 letters and a window size *w* = 30. This is just an illustration example, for the purpose of clarifying the explanation of the proposed method (these are not the optimal parameters for our problem).

The symbolic sequence should retain the important information of the original signal (Daw et al., 2003). However, not all information will pass from the real-valued ECG to the symbolic ECG, since, in this process, there is loss of information, and it is essential to guarantee that the information loss does not compromise the ECG information. The mutual information, the Pearson correlation and the Euclidean distance between the original and the quantized signal, were calculated. Because the quantized signal cannot be directly compared with the original ECG, the quantized signal should be reconstructed, in this case by means of a cubic spline.

For the optimization of the parameters of the method, the objective function was defined as the maximization of both the mutual information and Pearson's correlation, and the minimization of the Euclidean distance. The parameters were varied between 10 and 20 sample points, and the alphabet size between 2 and 20 symbols.

#### 2.3.2. The normalized relative compression measure

One of the most interesting and important problems that can be addressed using the Kolmogorov complexity theory is the definition of a similarity measure between binary objects. In this context, Li et al. proposed a similarity metric (Li et al., 2004) based on an information distance (Bennett et al., 1998), defined as the length of the shortest binary program that is needed to transform *x* and *y* into each other. This distance depends not only on the Kolmogorov complexity of *x*, *K*(*x*), and *y*, *K*(*y*), but also on conditional complexities (e.g., *K*(*x*|*y*), that indicates how complex is *x* when *y* is known). Because this distance is based on the Kolmogorov complexity (non-computable), they proposed a practical analog based on standard compressors, called the normalized compression distance (Li et al., 2004).

In order to approximate the Kolmogorov complexity, a compression algorithm needs to be able to accumulate knowledge of the data while the compression is performed. It has to find dependencies, to collect statistics, i.e., it has to create an internal model of the data. Finite-context models (FCMs) possess those properties and have been successful used in data modeling, such as DNA sequences and images, usually in the context of data compression (Pinho et al., 2011; Matos et al., 2013). Recent work has demonstrated their ability to create similarity/dissimilarity measures that rely on the data algorithmic entropy (Pinho and Ferreira, 2011; Pratas et al., 2012; Garcia et al., 2013).

The specific measure that we used is based on the concept of *relative compression* of two objects, denoted by *C*(*x*||*y*), i.e., the compression of object *x* is performed using *only* information available from another object, *y*. We define the normalized relative compression (NRC) of *x* given *y* as:

(1) $$\text{NRC}(x\|y)=\frac{C(x\|y)}{\left|x\right|},$$

where |*x*| is the size of the object *x*. Basically, this measure gives information about the fraction of data in *x* that cannot be described efficiently by *y*. Therefore, *x* is encoded exclusively using the model built from *y*.

Our implementation of *C*(*x*||*y*) is based on a combination of FCMs (Bell et al., 1990) of several orders, *k*, that are used to build an internal model of *y*, kept fixed afterwards. For that purpose, we developed an encoder that adaptively mixes the probability estimates of the several FCMs. The mixture weights are continuously adapted during compression, according to the performance of each individual FCM.

A FCM complies to the Markov property, i.e., it estimates the probability of the next symbol of the information source using the *k* > 0 immediate past symbols (order-*k* context) to select the probability distribution. Therefore, assuming that the *k* past outcomes are given by $x_{n-k+1}^{n}=x_{n-k+1}\cdots x_{n}$, the probability estimates, $P\left(x_{n+1}|x_{n-k+1}^{n}\right)$, are calculated using symbol counts that are accumulated while the information source is processed, with:

(2) $$P(s|x_{n-k+1}^{n})=\frac{\nu (s|x_{n-k+1}^{n})+\alpha }{\nu (x_{n-k+1}^{n})+\alpha |A|},$$

where $A=\left\{s_{1},s_{2},\ldots ,s_{\left|A\right|}\right\}$ is the alphabet that describes the objects of interest, $\nu \left(s|x_{n-k+1}^{n}\right)$ represents the number of times that, in the past, symbol s∈A was found having $x_{n-k+1}^{n}$ as the conditioning context and where:

(3) $$\nu (x_{n-k+1}^{n})=\sum_{a\in A}\nu (a|x_{n-k+1}^{n})$$

denotes the total number of events that has occurred within context $x_{n-k+1}^{n}$. The parameter α allows balancing between a maximum likelihood estimator and a uniform distribution. Notice that when the total number of events, *n*, is large, (2) behaves as a maximum likelihood estimator.

After processing the first *n* symbols of *x*, the average number of bits generated by an order-*k* FCM is:

(4) $$H_{k,n}=-\frac{1}{n}\sum_{i=1}^{n}\log_{2}P(x_{i}|x_{i-k}^{i-1}),$$

where we assume the convention that $x_{1-k}^{0}$ is known to both the encoder and decoder. *H*_*k,n*_ can be viewed as a measure of the average performance of model *k* until position *n*. Therefore, the overall probability estimate for position *n* + 1 can be given by the weighted average of the probabilities provided by each model, according to their individual performance, i.e.:

(5) $$P(x_{n+1})=\sum_{k\in K}P(x_{n+1}|x_{n-k+1}^{n})\;w_{k,n},$$

where K denotes the set of |K| models involved in the mixture, and:

(6) $$w_{k,n}=P(k|x_{1}^{n}),$$

i.e., the weights correspond to the probabilities that each model has generated $x_{1}^{n}$. Hence, we have:

(7) $$w_{k,n}=P(k|x_{1}^{n})\propto P(x_{1}^{n}|k)P(k),$$

where $P\left(x_{1}^{n}|k\right)$ denotes the likelihood of sequence $x_{1}^{n}$ being generated by model *k* and *P*(*k*) denotes the prior probability of model *k*. Assuming P(k)=1/|K|, we also obtain:

(8) $$w_{k,n}\propto P(x_{1}^{n}|k).$$

Calculating the logarithm of this probability we get:

(9a) $$\log_{2}P(x_{1}^{n}|k)=\log_{2}\prod_{i=1}^{n}P(x_{i}|k,x_{1}^{i-1})=$$

(9b) $$=\log_{2}\prod_{i=1}^{n}P(x_{i}|x_{i-k}^{i-1})=\sum_{i=1}^{n}\log_{2}P(x_{i}|x_{i-k}^{i-1}),$$

which is related to the number of bits that would be required by model *k* to represent the sequence $x_{1}^{n}$. Therefore, it is related to the accumulated measure of the performance of model *k* until position *n*.

To facilitate faster adaptation to non-stationarities of the data, instead of using the whole accumulated performance of the model, we adopt a progressive forgetting mechanism. The idea is to let each model to progressively forget the distant past and, consequently, to give more importance to recent performance results. To accommodate this, we first rewrite (9) as:

(10) $$\log_{2}P(x_{1}^{n}|k)=\sum_{i=1}^{n-1}\log_{2}P(x_{i}|x_{i-k}^{i-1})+\log_{2}P(x_{n}|x_{n-k}^{n-1})$$

and then

(11) $$\log_{2}p_{k,n}=\gamma \log_{2}p_{k,n-1}+\log_{2}P(x_{n}|x_{n-k}^{n-1}),$$

where γ ∈ [0, 1) is the forgetting factor and (−log_2_*p*_*k,n*_) represents the estimated number of bits that would be required by model *k* to represent the sequence $x_{1}^{n}$ (we set *p*_*k*,0_ = 1), taking into account the forgetting mechanism. Removing logarithms, we rewrite (11) as:

(12) $$p_{k,n}=p_{k,n-1}^{\gamma }P(x_{n}|x_{n-k}^{n-1})$$

and, finally, we set the weights to:

(13) $$w_{k,n}=\frac{p_{k,n}}{\sum_{k\in K}p_{k,n}},$$

to enforce normalization.

### 2.4. Biometric vs. emotion identification

**Figure 2:**
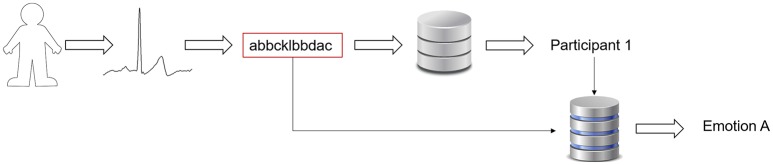
Algorithm workflow representation.

The NRC measure was used as a dissimilarity measure, which evaluates if two ECG segments are closer, i.e., in the case of two ECG segments coming from the same source (person or emotion), it is supposed to obtain a lower value, comparing with the NRC for ECG segments coming from different sources. To compute the measure, at first we should define the template, which depends on the hypothesis. If we want to evaluate the emotion, the template should comprise ECG segments that represent that emotion. If we want to evaluate the biometric identification, the template should represent the person that we want to identify. Figure 2 presents the workflow used in order to identify both the person and the emotion.

Considering the biometric identification, and in order to characterize the participants in the database, one template was designed for each participant in the database. The idea is to have a representation for each person in the database. In the presence of a new record, it will be compared with each participant template and interpret to whom the new record corresponds.

### 2.5. Statistical analysis

We adopted a leave-one-out strategy for the evaluation of the method. On the PTB database, four segments from each participant constituted the training dataset and the remaining segment was used as the test dataset. The proposed method was evaluated in two steps:

1. The results were presented as mean ± standard deviation of the calculated measure (NRC) between records. The results were aggregated as self-evaluation (NRC value comparing the record with itself), participant measure (NRC value calculated between records of the same participant, excluding self-similarity), out measure (NRC value calculated between records of different participants).
2. A 1-NN classifier was applied, considering as distance measure the proposed method in this paper (the NRC value). The idea was to assess the identification performance, which was evaluated by means of sensitivity (Equation 14), specificity (Equation 15) and accuracy (Equation 16):
  (14) $$sen=\frac{TP}{TP+FN},$$
  (15) $$spec=\frac{TN}{FP+TN},$$
  (16) $$acc=\frac{TP+TN}{TP+FP+TN+FN},$$

where *TP* is the number of true positives, *TN* the number of true negatives, *FP* the number of false positives and *FN* is the number of false negatives. The sensitivity represents the ability of a classifier to identify someone correctly. The specificity evaluates the ability of the classifier to reject someone correctly. The accuracy gives the proportion of true (both true positive and negative) results over all possible cases. The proposed method was tuned using the PTB database. Then, it was validated over a different database, the emotional database. The idea behind the use of this last database was to evaluate the performance of the method, considering the recognition rate, in both biometric and emotion identification, regarding different data collection conditions, thus evaluating the reproducibility of the method.

## 3. Results and discussion

Both biometric and emotion identification have a signature over the ECG. The inter-variability between persons allows an intrinsic key on the ECG. The NRC was used as a dissimilarity/similarity measure. In this work we tested the hypotheses that the mutual information contained in two segments from the same source may be quantified using compression methods, i.e., when two segments—A and B—come from the same source, we need less information to describe B when A is given. This evaluation will be used as a quantification process of similarity between files.

### 3.1. Biometric identification using the PTB database

As a first step validation, the PTB database (Goldberger et al., 2000) was used in order to tune the model parameters, validate the model, and compare the obtained results with other similar methods already published. Our method is composed by two steps: the quantization and the compression. In the quantization step, it is important to correctly optimize the model parameters. It is known that, when quantization is performed, there is loss of information. However, it is crucial that this loss of information does not compromise the ECG information. Therefore, and in order to quantify the information loss, three statistics were calculated: the mutual information, the Euclidean distance, and the Pearson correlation between the original and the reconstructed ECG (by means of a cubic spline) after quantization. There is not a rule to find out these parameters, so we tested a combination of parameters and evaluated the identification performance (Bras and Pinho, 2015). The best results were associated to an alphabet size of 20 and a window size of 3. Considering these parameters, the mutual information was 0.85 ± 0.11, the Euclidean distance was 8.60 ± 0.51, and the Pearson correlation was 0.90 ± 0.04.

Considering the optimal parameters found in the previous step, all ECG signals were transformed into its corresponding symbolic sequences, allowing the application of the NRC measure. Nevertheless, to apply the NRC measure, it is also needed the optimization of three parameters: γ, α, and *k* (Bras and Pinho, 2015). In our NRC configuration, a γ of 0.2 and a combination of (α, *k*) pairs taken from set {(1/1, 000, 5), (1/1, 000, 6), (1/1, 000, 7), (1/1, 000, 8), (1/1, 000, 9)} were used. As objective function in the model performance evaluation, it was used the minimization of the self-evaluation (comparing the record with itself). In Table 1, the identification results are presented. The NRC was able to differentiate between the three data groups, i.e., the NRC clusters the data in three groups or training segments, same or different participant:

1. ECG training segments (with a mean value of 0.150);
2. ECG testing segment from the same participant (with a mean value of 0.342);
3. ECG testing segments from different participants (with a mean value of 0.568).

**Table 1:** The NCR measure results summarized on train, test and global datasets.

**Dataset**	**Self-evaluation**	**Participant measure**	**Out measure**
Train	0.149 ± 0.096	0.325 ± 0.087	0.563 ± 0.102
Test	0.151 ± 0.104	0.346 ± 0.099	0.568 ± 0.135
Global	0.150 ± 0.103	0.342 ± 0.097	0.568 ± 0.135

*The results are presented as mean±std of the calculated measure*.

Because the proposed method should be validated in terms of identification performance, a 1-NN classifier was implemented. The classifier attained a sensitivity of 90.77% ± 9.97% (the sensitivity values distribution is not symmetric), a specificity of 99.96% ± 0.04% (the specificity values distribution is not symmetric), and a global accuracy of 99.93% ± 0.08%. The method proposed by Coutinho et al. (2013), which is also based on information theoretic models and was evaluated on the PTB database, attained an average recognition rate of 99.85% and an accuracy of 99.39%.

There are differences in the evaluation process followed by each of these two methods. In their paper, Coutinho et al. (2013) used 3 min of information, whereas in our model we used only approximately 1.5 min. Although the accuracy in our biometric system is higher than the one presented by Coutinho et al. (2013), the sensitivity is lower. Nevertheless, and considering the system security, it is generally preferable that a system rejects more efficiently than it accepts. The results of the two methods are not completely comparable, since the use of the PTB database is different, although in terms of accuracy the two methods are indeed comparable.

### 3.2. Biometric identification using the emotional database

The previously presented results aimed at evaluating the performance of the proposed method in a freely available database, allowing the comparison of results with previously published works. However, the PTB database does not have data collected in different days or considering different emotional conditions. Therefore, we decided to test the method in a more realistic database. The results obtained in the emotional database evaluate the ability of the method in the recognition of a person, independently of the day (since there are differences between days), hour (since there is a circadian variability on ECG), and emotion (since different emotions may induce different responses on ECG, e.g., increase in HR). Although we started by using a small set of basic emotions, future studies should include further ones in order to provide a wider range of recognition patterns.

The same model parameters, optimized using the PTB database, were used in this step. As a reference template, we considered 7 segments of 60 s from each of the three different records, for each participant. The test database is composed by all other ECG segments. Since the stimuli videos that each participant was exposed had different intensities of emotional content, it is expected that the ECG reacts accordingly, i.e., there will be alterations in the signal that prints a new mark that the NRC method should be able to overpass to identify the key of each participant besides the emotional content.

Considering the biometric identification results, we obtained a global recognition rate of 98.03%. Considering the train segments (first 7 segments in the three records), we obtained a recognition rate of 100%, while evaluating the method in the remaining segments the recognition rate decreased to 97.6%. We also observed differentiated results in different participants or emotions, with the lowest values being reported in fear and disgust conditions on test dataset (76.5%), and highest values corresponding to 100% match, which was observed in several participants, emotions or dataset. Table 2 reports the results obtained.

**Table 2:** Recognition rate in biometric identification considering the emotional database.

	**All database**				**Train**				**Test**			
	**Global**	**Fear**	**Neutral**	**Disgust**	**Global**	**Fear**	**Neutral**	**Disgust**	**Global**	**Fear**	**Neutral**	**Disgust**
Average	98.3%	98.0%	98.9%	98.0%	100.0%	100.0%	100.0%	100.0%	97.6%	97.2%	98.5%	97.2%
Std	2.2%	4.2%	2.8%	4.5%	0.0%	0.0%	0.0%	0.0%	3.0%	5.9%	3.8%	6.4%

(P belongs to participant and Std to standard deviation.)

These results show that, in the presence of different emotions, the method is also able to extract and identify the key features of the ECG, in order to identify the person. Because, in this case, the template used as reference contains examples of several emotions, the alteration that a particular emotion causes on the ECG does not blur the essential information. Obviously, the richer the reference template is in terms of intra-subject variability, the more robust will be the identification.

In order to try mimicking real conditions, we run a test where we used 7 segments of 60 s (following the previously described approach) from two ECG records collected in two different days and as target template we used data from the ECG left out from the reference. In this case, the performance reduces to values around 72%, because of the lower difference of the NRC similarity between the same participant (0.344 ± 0.134) and a different participant (0.489 ± 0.134). This result indicates that our method is still able to identify the person. However, it also shows that it is needed to have a database as rich as possible, regarding the representation of each participant, in order to improve the identification performance.

The method proposed in this work does not have as goal the extraction of features from the signal. Consequently, it is not possible to infer the steady characteristics in each emotion. The method compares the ECG records, by means of a compression algorithm, and uses the number of bits needed to describe a record using the information from the reference templates, in order to evaluate its similarity to the already known records. Therefore, the method does not perform an explicit feature extraction step. We argue that the intrinsic characteristics of each individual are in the ECG, independently of any external condition, and, hence, the data compression algorithms are able to find the regularities and use them to produce less bits to describe records from the same participant.

### 3.3. Emotion identification

Beyond the person identification (biometric identification), in this work we intended to extract more information from the ECG, specifically the emotional state of the identified person. This identification, integrated in daily routine and using non-intrusive systems, will enable to adapt systems, platforms, interactive homes, etc. The emotion defines more than who we are—it defines also what we are and what we want. By answering these questions, the environment surrounding us can adapt, increasing our comfort.

The defined template for emotion identification used the first 20 ECG segments from each participant. The performance of the method was evaluated over all dataset, where the last four ECG segments in each record/emotion were used as test templates. Again, the lowest NRC value (highest similarity) comparing a new ECG segment with the reference template indicated the classified emotion.

By inspection of Table 3, it is evident that the highest recognition rate is associated with the training dataset, and the lowest recognition rate is associated with the test dataset (as already expected). Even so, in the test dataset we obtained 77% in the fear detection, 74% in the neutral detection and 63% in the disgust detection.

**Table 3:** Recognition rate on emotion classification.

	**Global**			**Train**			**Test**		
	**Fear**	**Neutral**	**Disgust**	**Fear**	**Neutral**	**Disgust**	**Fear**	**Neutral**	**Disgust**
Fear	96.00%	4.67%	4.00%	99.80%	1.80%	0.00%	77.00%	19.00%	24.00%
Neutral	1.83%	93.83%	2.17%	0.00%	97.80%	0.00%	11.00%	74.00%	13.00%
Disgust	2.17%	1.50%	93.83%	0.20%	0.40%	100.00%	12.00%	7.00%	63.00%

*The results should be read in the vertical direction, e.g., 96.00% of the fear segments were classified as fear, 1.83% were classified as neutral and 2.17% were classified as disgust*.

The emotion is dependent on the person (Barrett et al., 2007; Kim and André, 2008; Agrafioti et al., 2012), therefore it is essential to identify the person first. We show that the same method is able to answer two questions. The template (reference) alteration guarantees that the focus of the method is different, i.e., when the template represents the records from the same person, it will identify the person. When the template is considering information from one specific emotion from one person, it will identify the emotion of that person. With this approach, we found that the biometric identification configuration is not affected by the alteration in emotion. However, we found evidence that the emotion identification is person dependent.

## 4. Conclusion

In this work, we proposed a method able to identify the person and the emotion using solely the ECG. Consistent with what has already been reported in the literature (Agrafioti and Hatzinakos, 2009; Agrafioti et al., 2012; Odinaka et al., 2012), we reinforced the conclusion that the ECG contains information able to represent the person individual characteristics. The proposed method is flexible and may, therefore, be adapted to different problems, by modifying exclusively the templates for training the model (they should be representative of the class that we want to identify).

Our method comprises three steps: (1) The quantization process, which allows to convert the real-valued ECG into a symbolic time-series; (2) The compression, using FCMs, and the quantification of the information needed to represent one record taken into account solely the template record, by means of the NRC; (3) The classification step using a 1-NN classifier.

The Normalized Relative Compression measure adequately compares two ECG segments, quantifying their similarity, which allows to stratify between groups. Using a 1-NN classifier, it was verified that the accuracy is over 98%, considering both evaluated databases. The sensitivity of the classifier, in the PTB database, is near 90%, and, in the emotional database, close to 98%.

As future work, we are planning to study the influence of different stimulus in the biometric identification method. In addition, extending this research work to positive emotions is of special relevance to understand whether the biometric system is also able to identify emotions with a positive valence. Also, we intend to extend the method to real time scenario, identifying the person and evaluating his/ her emotion at the access point.

Considering emotion identification, the method adequately infers the emotion conveyed by the ECG segments. It is important to reinforce that, in the first step, we identify the person, and only then, we identify the emotion. This approach relates with the fact that each person has a unique emotional experience and, therefore, particular response patterns are elicited by different stimulus.

The emotion identification allows the adaptation of systems and machines to the person needs. Specifically, an adaptable system that can read/decode a person's emotions and help him/her regulate them may be developed. For instance, in children with Autism Spectrum Disorders, who have difficulties in managing their emotions, a system able to identify emotions would be of great help. This system could be helpful in categorizing valence (positive or negative) and the intensity of different emotions, which could then enable appropriate actions from the children's formal and informal caregivers to aid in the regulations of such emotions.

The proposed method allows the biometric and emotion identification with performances comparable to those that have been previously shown in the literature. Also, the method does not require the ECG wave delineation and, therefore, the preprocessing error is expected to decrease.

## Ethics statement

This study and protocol was carried out in accordance with the recommendations of University of Aveiro with written informed consent from all subjects. All subjects gave written informed consent in accordance with the Declaration of Helsinki.

## Author contributions

SB, JF, SS, AP contributed to the work that culminates on the present paper. All of the authors were involved in the conception or design of the work, in the revision of the manuscript and they all approved the final version. SB was responsible for the analysis of data and all the authors contributed to the interpretation of data. JF collected the data, under the supervision of SS. Both JF and SS were responsible for the protocol design.

### Conflict of interest statement

The authors declare that the research was conducted in the absence of any commercial or financial relationships that could be construed as a potential conflict of interest. The reviewer, MJ, and handling Editor declared their shared affiliation.
